# A lactate shuttle system between tumour and stromal cells is associated with poor prognosis in prostate cancer

**DOI:** 10.1186/1471-2407-14-352

**Published:** 2014-05-21

**Authors:** Nelma Pértega-Gomes, José R Vizcaíno, Jan Attig, Sarah Jurmeister, Carlos Lopes, Fátima Baltazar

**Affiliations:** 1 Life and Health Sciences Research Institute (ICVS), School of Health Sciences, University of Minho, Braga, Portugal; 2 ICVS/3B’s - PT Government Associate Laboratory, Braga/Guimarães, Portugal; 3 Department of Pathology, Centro Hospitalar do Porto, Braga, Portugal; 4 MRC Laboratory of Molecular Biology, Francis Crick Avenue, Cambridge Biomedical Campus, Cambridge, UK; 5 Uro-oncology Research Group, Cancer Research UK Cambridge Institute, University of Cambridge, Cambridge, UK; 6 Department of Pathology and Molecular Immunology, Institute of Biomedical Sciences Abel Salazar (ICBAS), University of Porto, Porto, Portugal; 7 School of Health Sciences, University of Minho, Campus de Gualtar, 4710-057 Braga, Portugal

**Keywords:** Monocarboxylate transporters, Cancer associated fibroblasts, Poor prognosis, Prostate cancer

## Abstract

**Background:**

In a malignant tumour, cancer cells are embedded in stromal cells, namely cancer-associated fibroblasts (CAFs). These CAFs are now accepted as important players in cancer dynamics, being involved in tumour growth and progression. Although there are various reports on the interaction between tumour and stromal cells, the clinico-pathological significance of this cross-talk is still largely unknown. In this study, we aimed to characterise the expression of key metabolic proteins involved in glucose transport, pyruvate/lactate shuttle system, glycolytic metabolism and fatty acid oxidation in CAFs and tumour cells in different stages of malignant transformation. We further aimed to contextualise the clinico-pathological significance of these protein expression profiles with reference to known prognostic indicators, including biochemical recurrence in pT stage.

**Methods:**

Prostate tissues were obtained from 480 patients with a median age of 64 years following radical prostatectomy with no previous hormonal therapy. Tissues were analysed for the expression of several key metabolism-related proteins in glands and surrounding fibroblasts by immunohistochemistry. Reliable markers of prognosis such as pT stage and biochemical recurrence were assessed for each case.

**Results:**

We observed that prostate cancer cells did not rely mainly on glycolytic metabolism, while there was a high expression of MCT4 and CAIX - in CAFs. This corroborates the hypothesis of the “Reverse Warburg effect” in prostate cancer, in which fibroblasts are under oxidative stress and express CAIX, an established hypoxia marker. We found that alterations in the expression of metabolism-related proteins were already evident in the early stages of malignant transformation, suggesting the continuing alteration of CAFs from an early stage. Additionally, and for the first time, we show that cases showing high MCT4 expression in CAFs with concomitant strong MCT1 expression in prostate cancer (PCa) cells are associated with poor clinical outcome, namely pT3 stage of the tumour.

**Conclusions:**

In summary, this work demonstrates for the first time the clinico-pathological significance of the lactate shuttle in prostate cancer. It also suggests that other alterations in CAFs may be useful prognostic factors, and further supports the use of MCT1/MCT4 as targets for PCa therapy.

## Background

It is well established that solid tumours, including prostate cancer, exist under fluctuating oxygen tension, during which they are intermittently exposed to hypoxia [1,2]. Under hypoxic conditions, tumour cells primarily use glycolysis for energy, producing lactate, which is expelled to the tumour microenvironment, allowing tumours to continue their glycolytic activity [3,4]. Recently, Sonveaux *et al.* showed that lactate, which is generally considered a waste product, is preferred over glucose by oxidative tumour cells as their primary energy source [5].

Monocarboxylate transporters (MCTs) have been shown to play an important role in various tumours [6]. However, since they facilitate the transport of lactate in and out of cells, their role in this stromal/epithelial cell symbiosis is also attracting interest. MCT1 is a high-affinity transporter and its expression seems to be regulated by multiple signalling pathways, micro-environmental parameters, changes in substrate concentration and pH [7]. MCT4 is a low-affinity transporter, which is abundant in highly glycolytic muscle cells and is one of the many target genes of hypoxia-inducible factor 1 alpha (HIF-1α) [8]. Other targets of HIF-1α include glucose transporter-1 (GLUT-1), the main transporter involved in glucose uptake [9,10]; lactate dehydrogenase V (LDHV), which is responsible for the conversion of pyruvate into lactate; pyruvate dehydrogenase kinase isozyme 1 (PDK1), which is responsible for the phosphorylation and consequent inactivation of pyruvate dehydrogenase (PDH); and carbonic anhydrase IX (CAIX), a hypoxia-related protein involved in pH regulation [11]. Alpha*-*methylacyl-CoA racemase (AMACR), pristanoyl-CoA oxidase (ACOX-3) and D-bifunctional protein (DBP), are also important fatty acid oxidation-related proteins in prostate cancer [12,13], and we also included them in our analysis.

The importance of a lactate shuttle system between cancer cells and surrounding stroma has been described in various tumour types [14-16], but its significance in prostate cancer is not clear [17,18].

In this study, we aimed to identify a metabolic interaction between CAFs and prostate cancer cells, by analysing the expression of key metabolism-related proteins in CAFs in relation to prostate cancer using prostate tissue samples. We also assessed the clinico-pathological significance of this expression to investigate a possible CAF signature for PCa progression.

## Methods

### Patient sample selection

Formalin–fixed paraffin embedded tissues from 480 prostate cancer patients were retrieved from the archives of the Department of Pathology of Centro Hospitalar do Porto, Portugal. Stroma surrounding non-neoplastic glands, prostatic intra-epithelial hyperplasia (PIN) lesions and malignant glands were also analysed. Prostate cancer patients were selected for the study according to the following criteria: availability of both tumour and normal tissue for each patient, presence of adequate amount of stroma in both normal and tumour tissues for efficient selection for tissue microarray construction (TMA), and absence of chemotherapy or radiotherapy. Prior to TMA construction, tissue morphology was assessed on HE slides. Data for clinical parameters significant in patient outcome were available, including pre-operative serum total PSA, clinical stage, perineural invasion and biochemical recurrence.

### Ethics

The work has been approved by DEFI (Departamento de Ensino Formação e Investigação) Ethics Committee of Centro Hospitalar do Porto ref. no. 017/08(010-DEFI/015-CES).

### Immunohistochemistry

Samples organised into TMAs including 203 non-neoplastic, 176 PIN and 480 neoplastic tissues were analysed for MCT1, MCT4, GLUT-1, GLUT-12, LDHV, PDK1, CAIX, AMACR, ACOX-3 and DBP expression. Staining was evaluated using a combined score system, as previously described [19]. Detailed information regarding the immunohistochemistry (IHC) technique is given in Table 1.

**Table 1:** Details of the immunohistochemical procedure used to analyze the expression of the different proteins

**Protein**	**Antibody**	**Company**	**Antibody dilution**	**Positive control**	**Incubation period**	**Detection system**
**MCT4**	sc-50329	Santa Cruz Biotechnology	1:500	Colon tumor	Overnight	R.T.U. Vectastain Universal Elite ABC Kit, Vector, EUA
**GLUT1**	ab 15309	Abcam	1:2000	Head and neck tumor	2 hours	Ultravision Detection System Anti-polyvalent, HRP, Labvision Corporation, Freemont, CA
**CAIX**	ab 15086	Abcam	1:2000	Stomach		
**MCT1**	sc-365501	Santa Cruz Biotechnology	1:500	Colon tumor	Overnight	R.T.U. Vectastain Universal Elite ABC Kit, Vector, EUA
**GLUT12**	ab 75441	Abcam	1:500	Kidney	Overnight	
**LDHV**	ab 53010	Abcam	1:1000	Colon tumor		
**PDK1**	ab 110025	Abcam	1:500	Stomach		
**AMACR**	504R-16	Cell Marque	1:50	Kidney		
**ACOX3**	sc-135435	Santa Cruz Biotechnology	1:250	Liver	2 hours	Ultravision Detection System Anti-polyvalent, HRP, Labvision Corporation, Freemont, CA
**DBP**	DBP antibody was a gift from Dr. Gabriele Moller from HelmholtzZentrum mÜnchen		Ready to use	Kidney		

### Immunohistochemical evaluation

IHC evaluation was performed as previously described [19] and scored independently by two pathologists (JRV, CL), blinded to the target under study. For statistical purposes, only moderate and strong immunoreaction final scores were considered positive. Discordant cases were discussed in order to agree on a final score.

### Statistics

**Figure 1:**
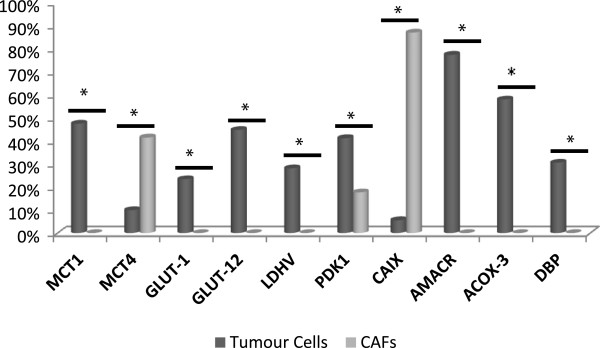
Comparison between metabolism-related proteins expression in tumour cells and cancer associated fibroblasts (CAFs).

Statistical analysis was performed using the SPSS statistical software (version 17.0, SPSS Inc., Chicago, IL, USA). All comparisons were examined for statistical significance using Pearson’s chisquare *(χ*^2^) test, using a threshold for significance of *p* < 0.05. Figure 1 was generated in part using R statistical computing environment, R version 3.0.0 [20] The code for the aesthetics of the stacked bar graphs was originally written by Kim Herzig [21].

## Results

We observed obvious differences between the expression of key metabolism-related proteins in CAFs and tumour cells (Figure 1). MCT1, MCT4, LDHV, PDK1, GLUT-1, GLUT-12, CAIX, AMACR, ACOX-3 and DBP were differentially expressed between stromal and epithelial cells, while MCT1, LDHV, GLUT-1, GLUT-12, AMACR, ACOX-3 and DBP were exclusively expressed in prostate cancer cells. MCT4 and CAIX were expressed more strongly in CAFs, and PDK1 stained both malignant glands and CAFs.

**Figure 2:**
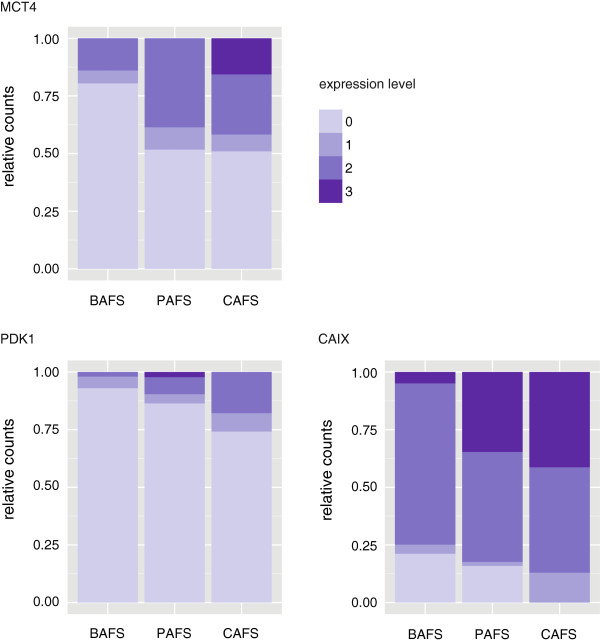
**Stacked bar graph according to one protein column within each fibroblast group for MCT4, PDK1 and CAIX expression.** The stronger expression (3) is represented by the more intense colour.

**Figure 3:**
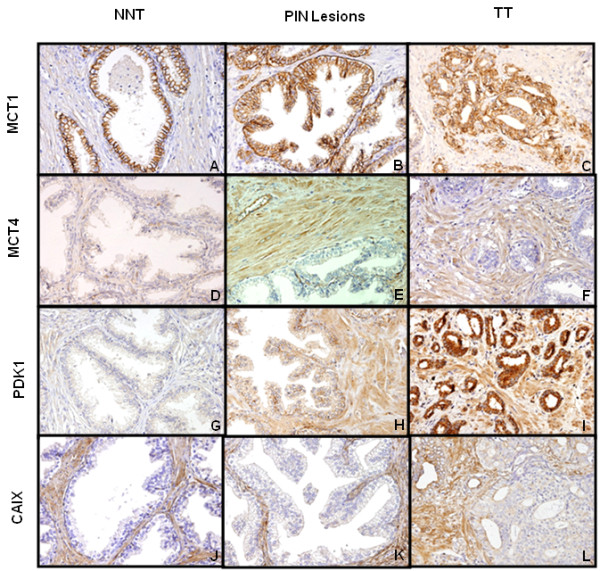
**Immunohistochemical staining for MCT1, MCT4, PDK1 and CAIX expression in non-neoplastic tissue (NNT), PIN lesions (PIN), tumour tissue (TT) and the surrounding stroma for each case.** Strong expression of MCT4, PDK1 and CAIX in stromal cells is evident, in contrast with MCT1, which is present only in the epithelial cells of the glands.

Additionally, we assessed whether fibroblasts exhibited differences in protein expression across different stages of malignant transformation by analysing the expression of the same proteins in fibroblasts surrounding benign glands (benign-associated fibroblasts; BAFs), PIN-associated fibroblasts (PAFs) and CAFs. Figure 2 shows stacked bar graphs representing the expression of MCT4, PDK1 and CAIX in more detail, since these proteins were the ones exhibiting a clear expression in fibroblasts surrounding both benign and malignant glands. A statistically significant increase in both MCT4 and PDK1 expression in CAFs compared to BAFs was observed (both p < 0.001). Curiously, CAIX was also observed in fibroblasts surrounding non-neoplastic glands (benign glands and PIN lesions) (see also Figure 3).

Key metabolism-related proteins in fibroblasts and prostate glands across malignant transformation, i.e. from BAFs to PAFs and finally CAFs, were investigated. MCT1 was clearly expressed in the plasma membrane of prostate glands, but not the surrounding stroma. In contrast, the expression of MCT4 increased in fibroblasts with increasing degree of malignant transformation, but not in the prostate glands. PDK1 expression was detected in both glands and stroma, whereas CAIX was only detected in stroma, with no staining in prostate glands (Figure 3).

Associations between the expression of the metabolic proteins and clinico-pathological data are presented in Table 2. We observed that CAIX expression in CAFs was associated with biochemical recurrence after surgery (*p* = 0.003). Furthermore, the strongest associations identified were in samples with elevated levels of MCT1 in tumour cells together with elevated levels of MCT4 in the surrounding CAFs. These cases were associated with pT3 tumour stage (*p* = 0.009). Additionally, cases negative for both MCT1 and MCT4, or positive for MCT1 in the malignant glands and negative for MCT4 in CAFs, showed no associations with clinico-pathological parameters (data not shown).

**Table 2:** Correlations between the key metabolic-related proteins MCT4, PDK1 and CAIX expressions in CAFs and clinico-pathological data

		**MCT4**		**PDK1**		**CAIX**		MCT1/MCT4*	
**Variable**	**n**	**%**	***p***	**%**	***p***	**%**	***p***	**%**	***p***
**PSA (ng/ml)**			0.296		0.451		0.250		0.525
≤ 5.0	245	47.9		16.2		85.9		27.1	
>5.0	123	44.0		17.5		82.2		27.5	
**pT**			0.445		0.258		0.522		**0.009**
2	359	40.2		18.7		86.4		20.8	
3	99	41.7		15.2		86.9		33.3	
**Perineural**									
**Invasion**			0.155		0.069		0.399		0.255
Absent	119	45.4		13.4		85.8		26.9	
Present	327	39.4		19.8		87.2		23.3	
**Biochemical**									
**Recurrence**			0.104		0.176		**0.003**		0.052
Absent	410	40.2		18.5		75.4		22.6	
Present	69	49.3		13.0		89.0		32.8	

The correlation between MCT1/MCT4 expression (*MCT1 expression in prostate tumour cells with concomitant expression of MCT4 in CAFs) and the clinico-pathological data is also presented.

## Discussion

Several research groups have recently focused on the role of CAFs in the progression and metastasis of prostate cancer, showing that a dynamic interaction between stroma and epithelium might play a critical role in this progression [14,15,22-25]. Thus, the essential role played by the cross-talk between stroma and epithelium in carcinogenesis and prostate cancer progression has been increasingly recognised. In this work, we provide evidence for the possible metabolic co-operation between cancer cells and the surrounding fibroblasts by examining the expression of major proteins involved in cellular metabolism. In particular, we focus on differences between cancer cells and tumour-associated fibroblasts, as well as between fibroblasts in different stages of malignant transformation, and examine the possible clinico-pathological significance of the expression of these proteins.

By categorising the protein expression of stromal cells associated with prostate cancer, we describe a compartment that is not well studied and will contribute to an improved understanding of prostate cancer. We observed significant differences between CAFs and tumour glands with respect to the expression of key metabolic proteins. In particular, CAIX and MCT4 selectively labelled cancer associated fibroblasts in contrast to malignant glands, where CAIX and MCT4 were only present in very few cases. On the other hand, a distinct, strong membranous expression of MCT1 was consistently observed in cancer cells, suggesting a role for MCT1 in the transport of lactate into tumour cells from the acidic extracellular matrix, suggesting that lactate might be used as a fuel by oxidative cancer cells. We also observed that proteins involved in fatty acid oxidation, such as AMACR, ACOX-3 and DBP, were restricted to the tumour cells, which is consistent with the presence of a metabolic pathway different from glycolysis, and compatible with oxidative phosphorylation in prostate cancer cells. It is important to note that fatty acid oxidation is already considered a major source of acetyl-CoA for the Krebs cycle [13], which further supports our hypothesis.

The expression levels of GLUT1, a key glucose transporter, define the rates of glucose influx into the cells. In the present study, CAFs did not show GLUT1 or GLUT-12 expression, and LDHV was also difficult to detect. However, this possibly reflects the limits of the immunohistochemical technique to detect these proteins at the baseline concentrations present in CAFs. Indeed, we have previously found very few cases positive for GLUT-1 and GLUT-12, and this expression was not present at the plasma membrane, suggesting a low level of activity of these proteins in prostate cancer cells (unpublished results). Thus, assessment of other GLUT isoforms may be worthwhile.

Interestingly, we also observed that protein expression of MCT4, PDK1 and CAIX in prostate fibroblasts changes during malignant transformation, suggesting that the existing stroma might also suffer alterations and play a role in this metabolic adaptation of cancer cells beyond the well-studied role of newly formed stroma.

From the above immunohistochemical findings, it seems that well-organised metabolic regions composed of tumour cells and CAFs may contribute to the ability of the tumour to overcome the adverse microenvironment.

Our hypothesis is in agreement with those of Fiaschi *et al.*[17], who describe the metabolic reprogramming of CAFs towards the Warburg phenotype as a result of contact with prostate cancer cells. Using *in vitro* studies, they showed lactate production and efflux by *de novo* expressed MCT4 in CAFs and also demonstrated that, upon contact with CAFs, prostate cancer cells were reprogrammed towards aerobic metabolism, with an increase in lactate uptake via the lactate transporter MCT1. Furthermore, pharmacological inhibition of MCT1-mediated lactate uptake dramatically affected PCa cell survival and tumour outgrowth. However, in this study, no data regarding clinico-pathological associations were shown, and few cases were assessed. These findings are in contrast with others ([18], which describe an energy recycling path between the aerobic stroma and the anaerobic cancer cells within the framework of the Warburg effect. These conclusions are based mainly on the observation that LDH1 is evidently expressed in CAFs, and the presence of MCT1 in prostate cancer cells was attributed to its role in lactate efflux and not its uptake. We recognise the importance of assessing LDH1; however, in our study we assessed for the first time MCT4 and CAIX as important markers of hypoxia in a larger cohort. Our findings corroborate the work of Whitaker-Menezes *et al.*[16], who described a “reverse Warburg effect,” where CAFs undergo aerobic glycolysis to produce lactate, which is subsequently used as a metabolic substrate by adjacent cancer cells. In this model, “energy transfer” or “metabolic coupling” between the tumour stroma and epithelial cancer cells fuels tumour growth and metastasis via oxidative mitochondrial metabolism in anabolic cancer cells. We believe that this is also the case in prostate cancer, although more studies are needed to demonstrate this.

Also, we assessed important clinico-pathological parameters and found significant associations with poor prognosis, raising once more the possible role of CAFs in disease management. We believe that these changes are likely to be a by-product of tumour biology with further influence on patient outcomes that need to be explored more deeply.

**Figure 4:**
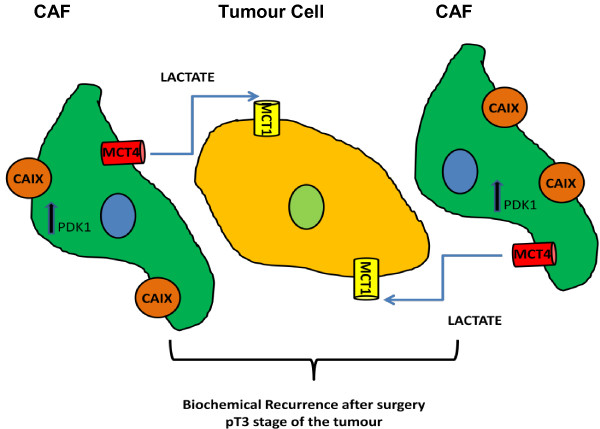
**Schematic representation of the lactate shuttle system between malignant cells and cancer associated fibroblasts (CAFs).** The expression of MCT4 in CAFs together with the expression of MCT1 in tumour cells is associated with biochemical recurrence after surgery and pT3 stage of the tumour.

In summary, we found differences between prostate cancer cells and CAFs using tissues from 480 patients, showing elevated expression of MCT4 and CAIX in CAFs and demonstrating for the first time that the concomitant expression of MCT1 in tumour cells and MCT4 in fibroblasts in the same tissue is clinically significant, and associated with poor prognosis. Indeed, the stromal expression of hypoxia-regulated proteins appears to be prognostic of poor outcome in prostate carcinomas, suggesting that tumour hypoxia may influence tumour-associated stromal cells in a way that ultimately contributes to patient outcome. Figure 4 shows a schematic representation of the lactate shuttle between CAFs and PCa cells to illustrate the hypothesis presented in our work.

## Conclusions

In summary, we show for the first time that there is a clinico-pathological significance for the MCT1/MCT4 lactate shuttle in prostate cancer. In fact, it seems that the stromal expression of hypoxia-regulated proteins is an adverse prognostic factor in prostate carcinomas, suggesting that tumour hypoxia may influence tumour-associated stromal cells in a way that ultimately contributes to patient prognosis.

## Competing interests

The authors declare no competing interests.

## Authors’ contributions

NPG and FB were responsible for the study concept and design, manuscript drafting and critical revision. NPG carried out the experiments and was responsible for sample and clinico-pathological data collection. JRV and CL evaluated the immunohistochemical reactions. JA and SJ were involved in figures generation. All the authors read and approved the final manuscript.

## Pre-publication history

The pre-publication history for this paper can be accessed here:

http://www.biomedcentral.com/1471-2407/14/352/prepub
